# The Key Enzymes of Carbon Metabolism and the Glutathione Antioxidant System Protect *Yarrowia lipolytica* Yeast Against pH-Induced Stress

**DOI:** 10.3390/jof10110747

**Published:** 2024-10-29

**Authors:** Tatyana I. Rakhmanova, Natalia N. Gessler, Elena P. Isakova, Olga I. Klein, Yulia I. Deryabina, Tatyana N. Popova

**Affiliations:** 1 Department of Medical Biochemistry and Microbiology, Biology and Soil Science Faculty, Voronezh State University, Universitetskaya pl., 1, 394000 Voronezh, Russia; rtyana@mail.ru (T.I.R.); biomed-popova@yandex.ru (T.N.P.); 2 Research Center of Biotechnology of the Russian Academy of Sciences, A.N. Bach Institute of Biochemistry, Leninsky Ave. 33/2, 119071 Moscow, Russia; gessler51@mail.ru (N.N.G.); klein_olga@list.ru (O.I.K.); yul_der@mail.ru (Y.I.D.)

**Keywords:** *Yarrowia lipolytica*, environmental stress, extreme pH, Krebs cycle enzymes, aconitate hydratase, stress defense system

## Abstract

In this study, we first thoroughly assayed the response of the key enzymes of energy metabolism and the antioxidant system in *Yarrowia lipolytica* yeast at extreme pH. The activity of the tricarboxylic acid cycle enzymes, namely NAD-dependent isocitrate dehydrogenase, aconitate hydratase, NAD-dependent malate dehydrogenase, and fumarate hydratase, NADPH-producing enzymes of glucose-6-P dehydrogenase and NADP-dependent isocitrate dehydrogenase, and the enzymes of the glutathione system was assessed. All the enzymes that were tested showed a significant induction contrary to some decrease in the aconitate hydratase activity with acidic and alkaline stress. It is probable that a change in the enzyme activity in the mitochondria matrix is involved in the regulation of the cellular metabolism of *Y. lipolytica*, which allows the species to prosper at an extreme ambient pH. It distinguishes it from any other type of ascomycete. A close relationship between the induction of the Krebs cycle enzymes and the key enzymes of the glutathione system accompanied by an increased level of reduced glutathione was shown. The assumption that the increased activity of the Krebs cycle dehydrogenases and promotion of the pentose phosphate pathway at pH stress launches a set of events determining the adaptive response of *Y. lipolytica* yeast.

## 1. Introduction

Ambient pH is crucial to maintaining the normal functioning of yeast cells. The transmembrane potential of the cytoplasmic membrane provided by the intracellular and external pH ratio plays a key role in nutrient transport. So, the transport of glucose and some other sugars needs H^+^-dependent symporters [1], and so does the amino acid transport system [2]. In addition, the proton-bound transporters together with K^+^ transporters, Tr1 and Tr2 proteins, and Na^+^/H^+^ antiporters affect the cytosol pH, and maintaining a proton gradient is obligatory for their normal operation [3]. The optimal pH for yeast is reported to be 5.5 (the optimum pH for the cytoplasmic H^+^-ATPase activity of *Saccharomyces cerevisiae*) although it can vary significantly depending on the species [4].

In the yeast cell adaptation to external pH alterations, the acidic and alkaline stress responses are distinguished. In fungi strains, acid stress frequently accompanies the production of organic acids. Upon cultivation, fungal cells secrete protons accumulating in the culture medium that inhibit cell growth [5]. There are some reasons for this, including the alteration in the conformation of membrane proteins and the cell wall composition due to the external pH effect, as well as some changes in the lipid bilayer resulting from the action of hydrophobic cations of organic acids. Being exposed to these factors, the cytoplasmic membrane permeability for various ions increases, followed by the excessively charged compounds entering the cell with signaling properties (namely, both protons and single and divalent cations). Weak organic acids penetrating the hyperpolarized membranes are of special danger to the cells. In the cytoplasm where pH is significantly lower than outside the cell, the acids dissociate, thereby increasing the proton number inside the cells [6]. 

Some experiments on *S. cerevisiae* at acidic stress showed changes in numerous genes responsible for the structural organization of the cell wall, as well as metal metabolism. Using proteomics methods, it was manifested that no ATPase of the vacuoles responsible for pumping protons from the cytoplasm into the vacuole, as well as the proteins of MAPK HOG1 stress signaling pathway, reduced tolerance to acid stress [7]. An acidic pH alters gene expression, which is controlled by the transcription factors of Aft1p and Aft2p, i.e., iron reductases FRE1-3, FRE5, iron permease FTR1, siderophore transporters of ARN1 and 2, ferroxidase (FET3), and the proteins associated with the siderophores FIT1–FIT3 absorption [8,9]. This was obtained using the microarrays method. It may indicate that acidic stress changes the metabolism of metal. The acidic stress also induced the transcription factor of AFT1 (iron-regulated transcriptional activator AFT1). Moreover, Aft1, highlighted by GFP, localized in the nuclei of the cells grown upon lactic, acetic, and hydrochloric acid addition. The same phenomenon was observed in conditions of iron deficiency. There are two possible explanations for that in acidic stress conditions: (1) the cells import no iron; (2) the cells need the increased iron level. Iron metabolism is known to depend not only on the external iron concentration but also on the iron–sulfur cluster activity, mainly localized in the mitochondria of the yeast. The results of some studies show that the concentration of the proteins containing iron–sulfur clusters encoded by the genes of *LEU1* (3-isopropyl malate dehydratase) and *ACO1* (aconitate hydratase, mitochondrial) significantly decreases under acidic conditions [10]. Moreover, Aft1p has been reported to be involved in the diauxic shift and oxidative stress [11]. Unlike acidic stress, alkaline stress depolarizes the membrane that significantly hinders the cell transporter action [12]. For example, in *S. cerevisiae* grown under optimal conditions, the Na^+^/H^+^ antiporter Nha1 is active, and monovalent cations (Na^+^, Li^+^, and K^+^) are released from the cell via this pathway. The process is crucial for phosphate transport mediated by the Na^+^ ion gradient. Under neutral and alkaline conditions, a change in the proton gradient complicates the process. In alkaline conditions, to overcome the obstruction, the sodium transport in the yeast switches to an alternative antiporter of Na^+^/K^+^ ATPase Ena [13]. The antiporter action creates a sufficient gradient of monovalent cations to launch the Pho89 phosphate transporter. The regulation of the Ano 1 gene expression is performed via various signaling pathways, in particular, the Pal/Rim pathway [14].

The Pal/Rim signaling pathway is the most investigated pathway of pH-sensitive signal transduction in fungi. It has been thoroughly studied using the *Aspergillus nidulans* (Pal) and *S. cerevisiae* (Rim). In *A. nidulans*, the Pal pathway successively involves the PalH, PalI, PalF, PalC, PalA, and PalB proteins. Moreover, the components of some other pathways are also optionally involved in this one, for example, cellular transport proteins [15]. Activation of the signaling pathway by alkaline pH induces the proteolytic activation of the PacC/Rim101 transcription factor. PacC, in turn, undergoes two successive proteolytic cleavages, the first of which is pH dependent and promotes the signaling PalB [16]. Pal/Rim 21, a plasmalemma protein consisting of seven transmembrane domains, is reported to function as a pH receptor. PalH forms a complex with the arrestin-like Pal/Rim 8 protein, which is pH-dependently ubiquitinated under alkaline conditions and recruits ESCRT-I Vps23, thereby creating numerous attachment sites for downstream signaling components [17].

In addition, some decrease in the solubility of important trace elements, namely iron and copper, is another negative consequence of the pH increase in cells. Deletions of some genes involved in metal metabolism, namely *CCC2* (copper-transporting ATPase), *AFT1* (iron-regulated transcriptional activator AFT1), *FET3* (iron transport multicopper oxidase FET3), *LYS7* (Superoxide dismutase 1 copper chaperone), and *CTR1* (copper transport protein CTR1), decreased cell survival under the alkaline conditions [14]. However, the additional copies of the *FAT4* (low-affinity Fe^2+^ transport protein) and *CTR1* genes raised the survivability. The lifted concentrations of iron and copper ions increased the *S. cerevisiae* tolerance to alkaline pH [15]. Thus, the adaptation of yeast and fungal cells to ambient extreme pH concerns the key aspects of cellular physiology from transport processes to energy metabolism pathways. 

Traditionally, the adaptation mechanisms in *Y. lipolytica* yeast have been studied at the level of global transcription regulators (proteins of Rim101 and Rim9) [18]. However, recently Rim21 has been suggested as a sensor in the Rim101 pathway, which can detect extracellular alkalinization [19]. Rim21 forms a sensor complex with Dfg16 and Rim9. Dfg16 and Rim9 are required for the stability and localization of Rim21 on the plasma membrane [20]. Moreover, Rim21 can be promoted by an altered lipid asymmetry of the plasma membrane. Obara and Kamura [19] have suggested the hypothesis that Rim21 is capable of detecting individual signals, namely external alkalinization and changed lipid asymmetry. The authors found that external alkalinization inhibits translayer movement of the phospholipids between two sheets of the plasma membrane, leading to injury in the lipid asymmetry of the plasma membrane. Moreover, the effective adaptation of *Y. lipolytica* to alkaline conditions is supposed to appear due to its ability to substitute the proton-dependent metabolite transport system through the plasma membrane for a similar Na^+^-dependent system. The conclusions are founded on the studies of regulatory proteins but they have so far yet to be confirmed by the direct data on the changes in the amount and/or activity of the most abundant proteins in the *Y. lipolytica* cells upon shifting the ambient pH [21].

*Yarrowia lipolytica* is an extremophilic yeast capable of resisting not only extreme pH but also some other unfavorable conditions, namely, high temperature, osmotic pressure, reduced water activity, and exposure to heavy metal ions [22]. The unique features have made *Y. lipolytica* a prospective biotechnological object, which is well amenable to genetic engineering manipulations and thriving under different cultivation conditions. The unique and untypical ability of *Y. lipolytica* to develop in a wide pH range, including alkaline conditions (up to a pH of 9.5), has been actively studied for a long time [23]. The ability to grow in extreme pH conditions makes *Y. lipolytica* a high potential for using as a producer of various products since the alkaline pH is an additional barrier against culture contamination. Moreover, *Y. lipolytica* is widely used as a producer of organic acids [24], in particular, due to its high tolerance to acidic pH. Since *Y. lipolytica* is intensively studied mainly for practical application, there is little information about the changes in its gene expression, proteome, or metabolome in acid stress conditions. However, sufficient data have been accumulated on the producer’s ability to synthesize various organic compounds under acidic conditions [25]. Thus, the pH of 2.8–4.0 was optimal for succinic acid synthesis [26], itaconic acid [27], and citric acid [28].

The study of changes in the cell proteome upon growing in alkaline conditions (pH 8.5) compared to that in the slightly acidic ones (pH 5.5) proved an essential step in understanding the excellent alkalotolerance of *Y. lipolytica*. In our previous study [29], we showed that all the proteins with increased amounts compared to the others in the *Y. lipolytica* cells grown at alkaline pH are in a mitochondrial location and are strongly involved in energy metabolism. They are the enzymes of the mitochondrial matrix, namely, malate dehydrogenase and α-ketoglutarate dehydrogenase, the outer membrane proteins (porin VDAC, non-selective voltage-gated ion channel), and some components of the respiratory chain. Later, using *Y. lipolytica* W29 grown under alkaline conditions, among the proteins with the elevated amount, we found two folding proteins (YALI0C10230p and YALI0F02805p), glycolysis proteins (YALI0C06369p and YALI0F05214p), mitochondrial proteins (outer membrane porin YALI0F17314p and malate dehydrogenase YALI0D16753p), ribosomal protein of A0A1H6Q0M6, and the nucleotide synthesis component of YALI0F09229p [30]. In [31], the authors revealed some changes in the proteome of *Y. lipolytica* WSH-Z06 in acid stress conditions upon producing α-ketoglutaric acid. The proteins maintaining intracellular pH homeostasis were divided into some classes by their cell functions. ATP synthesis is supposed to be induced because of membrane hyperpolarization during cytoplasmic acidification, which increases the reactive oxygen species (ROS) generation followed by the induction of antioxidant components and chaperones to protect the cell from oxidative stress. The level of the VDAC porin in the cells also rose to induce the exchange of metabolites between cytosol and mitochondria. 

Thus, the key enzymes of energy metabolism, mitochondrial transport systems, and the cellular antioxidant systems are involved in the pH-induced response of the yeast cell. In this study, we present a thorough assay of the activities of some Krebs cycle enzymes, NADPH-producing enzymes, and the components of the glutathione antioxidant system of the *Y. lipolytica* yeast at extreme pH.

## 2. Materials and Methods

### 2.1. Yeast Strain and Culture Conditions

The yeast was cultured for 24 h to the early stationary growth stage at different ambient pHs at a temperature of 29 °C as described in [32]. The pH of the broth containing the buffer was monitored using a pH meter every time. The pH of the cultural medium was 5.5. For the culture purity, microscopy was used with an inverted BiOptiqUI-100 microscope (BiOptiq, Moscow, Russia) throughout the whole experiment.

### 2.2. Preparation of Cellular Homogenate

The cellular homogenate was obtained as described before [33] with some modifications. The yeast cells were disrupted at 0 °C with an ultrasonic disintegrator QsonicaQ500 (Farmacia, Stockholm, Sweden) using eight pulses for 90 s interrupted by cooling periods every 30 s.

### 2.3. Cellular NAD-Dependent Isocitrate Dehydrogenase (NAD-IDH) Activity

Cellular NAD-IDH activity was measured as it was described in [34]. The activity of NAD-dependent IDH was assayed in the medium containing 40 mmol tris-HCl buffer (pH 7.4); 1.0 mmol sodium isocitrate; 4 mmol MgCl_2_; 0.5 mmol NAD. The extinction was assayed on a spectrophotometer at a wavelength of 340 nm for 5 min after the homogenate application. Activity was expressed in enzymatic units or as specific activity. A unit of enzymatic activity (E) was taken as the amount of enzyme catalyzing the formation of 1 μmol of the reaction product per 1 min at a temperature of 25 °C. The IDH activity was calculated according to the following formula:

E = D × 2.0 × V/∆V × τ × 6.22

where D is the increase in optical density at 340 nm; 2.0 is the solution volume in the cuvette, mL; V is the total enzyme volume, mL; ∆V is the sample volume added, mL; τ is the assay time, min; 6.22 is the extinction coefficient corresponding to the absorption value given by 1 μmol of isocitrate, in 1 mL of the test mixture upon assessing on a spectrophotometer, when the measured layer is 1 cm thick.

### 2.4. Assay of Aconitate Hydratase (AH) Activity

AH activity was measured as it was described in [35]. The activity of AH was assayed in a 50 mM Tris-HCl buffer (pH 7.8) containing 4 mM citrate. The extinction was assayed at 235 nm in 3 min after the homogenate application. Activity was expressed in enzymatic units or as specific activity. A unit of enzymatic activity (E) was taken as the amount of enzyme catalyzing the formation of 1 μmol of the reaction product per 1 min at a temperature of 25 °C. The AH activity was calculated according to the following formula:

E = D × 2.0 × V/∆V × τ × 3.09

where D is the rise in optical density at 235 nm; 2.0 is the solution volume in the cuvette, mL; V is the total enzyme volume, mL; ∆V is the sample volume added, mL; τ is the assay time, min; 3.09 is the extinction coefficient corresponding to the absorption value given by 1 μmol of cis-aconitate, in 1 mL of the test mixture upon assessing on a spectrophotometer, when the measured layer is 1 cm thick.

### 2.5. Assay of the NAD-Dependent Malate Dehydrogenase (NAD-MDH) Activity 

The enzyme activity was assessed spectrophotometrically at 340 nm in the medium containing 50 mmol Tris-HCl (pH 7.5); 1.5 mmol oxaloacetate, 0.15 mmol NADH. The unit of enzyme activity was calculated using the following formula:

E = ΔD × Vc × V/ΔV × τ × 6.22,

where ΔD is the decrease in A340 for a certain period; Vc is the volume of solution in the cuvette, mL; V is the total volume of enzyme solution, mL; ΔV is the amount of the sample, mL; τ is the time of measurement, min; 6.22 is extinction coefficient, the change in absorbance at 340 nm upon reduction or oxidation of 1 µmol coenzyme [36].

### 2.6. Assay of Fumarate Hydratase (FH) Activity 

The assessment of the FH activity is based on either a decrease (substrate –fumarate) or an increase (substrate –malate) in absorbance at a wavelength of 240 nm due to forming a double bond in the fumarate molecule. 20 µL sample was added to a 2 mL aliquot of incubation medium containing 50 mmol/L phosphate buffer (pH 7.4) and 5 mmol/L malate. The concentration of fumaric acid was assayed spectrophotometrically using absorption at 240 nm. For the enzyme activity, a general formula was used:

E = ΔD × Vc × V/ΔV × τ × 4.81,

where ΔD is the increase in D at 235 nm over a certain time; 2.0 is the volume of the solution in the cuvette, mL; V is the total volume of the enzyme solution, mL; ΔV is the volume of the sample introduced for measurement, mL; τ is the measurement time, min; and 4.81 is the extinction coefficient corresponding to the absorption, which is given by 1 µmol of fumarate in 1 mL of the test mixture upon measuring on a spectrophotometer, the layer of the measured solution is 1 cm thick [36].

### 2.7. Assay of Glucose-6-Phosphate Dehydrogenase (G6PDH) Activity

G6PDH activity was measured in 0.05 mM Tris-HCl-buffer, pH 8.0; containing 1 mM 6-phosphogluconate and 0.12 mM NADP^+^ by measuring the increase in absorbance resulting from the reduction of NADP^+^ at 340 nm. One unit of enzyme (EU) activity is that amount of enzyme, which reduces 1 µmol NADP^+^ per min at +25 °C. The calculation is the same as that for NAD-IDH [37].

### 2.8. Cellular NADP-Dependent Isocitrate Dehydrogenase (NADP-IDH) Activity 

NADP-IDH activity was assayed in a medium containing 0.05 mol/L Tris-HCl buffer (pH 7.8), 1.5 mmol isocitrate, 2 mmol MnCl_2_, and 0.25 mmol NADP. The reaction rate was assessed by the increase in the optical density of the test samples due to NADP reduction upon the enzyme-catalyzed conversion of isocitrate to 2-oxoglutarate [36].

### 2.9. The Free Radical Processes Assay Using the Induced Bio Chemiluminescence (BCL)

To assay the free radical processes intensity, we used bio chemiluminescence with a bio-chemiluminometer BHL-07 using hydrogen peroxide and iron sulfate. The method is based on the presented scheme, i.e., the catalytic decomposition of peroxide with metal ions of Fe^2+^ transition valence in the Fenton reaction. The formed free radicals (R*, OH*, RO*, RO_2_*, O_2_*) enter the initiation of free radical oxidation in the tested biological substrate. The recombination of RO_2_* radicals causes an unstable tetroxide, which decays and releases a quantum of light. The kinetic curve of bio-chemiluminescence was recorded for 30 s (the time of the maximal information about the free radical process intensity) and the following parameters, namely the light sum of chemiluminescence (S), flash intensity (I_max_), and the tangent of the angle of the curve inclination (tgα), describe the total antioxidant activity [38]. The method permits the assay of the free radical process in the organism. The medium contained 0.4 mL of 0.02 mM potassium phosphate buffer (pH 7.5), 0.4 mL of 0.01 mM FeSO_4_, and 0.2 mL of 2% hydrogen peroxide solution (introduced just before measurement). The 0.1 mL tested sample is applied before hydrogen peroxide addition and launches the reaction.

### 2.10. Assay of Enzymes Activities of Glutathione Antioxidant System

#### 2.10.1. Assay of Cell Glutathione Peroxidases (GPxs) 

GPxs in the cells were assessed at the wavelength of 340 using a Shimadzu RF 5301 PC spectrophotometer (Shimadzu, Tokyo, Japan). The 0.05 mM K-P_i_-buffer for measurement contained 1 mM EDTA, 0.12 mM NADP^+^, 0.85 mM reduced glutathione (GSH), 0.37 mM H_2_O_2_, and 1 unit of glutathione reductase (GR) per mL. For the control, the same medium without glutathione was used. The enzyme launched the reaction. The decrease in A showed NADP^+^ oxidation resulting in the reduction of GPxs due to its action. The enzyme amount catalyzing a 1 µmol final reaction product at +25 °C per a min defined one enzyme unit (EU) [39]. 

#### 2.10.2. GR Activity in the Cell

Cell GR activity was assayed at the wavelength 340 nm using a Shimadzu RF 5301 PC spectrophotometer (Shimadzu, Tokyo, Japan). Glutathione reduction was monitored by GR resulting in NADPH oxidation and the decrease in the absorbance. An amount of 0.05 M K-Pi-buffer contained 1 mM EDTA, 0.16 mM NADPH, 0.8 mM oxidized glutathione (GSSG), and pH 7.4. Cell suspension (1–5 × 10^8^ cells) was applied to 1 mL of the buffer. The calculation is the same as that for NAD-IDH [37].

### 2.11. Assay of GSSG and GSH

Yeast cells were disrupted in liquid nitrogen, and transferred to 0.1 M potassium phosphate buffer; pH 8.0, with a ratio of 1:5. Adding an equal volume of 5% metaphosphoric acid (Reachim, Moscow, Russia) and keeping the mixture on ice for 20 min precipitated the proteins. Next, the extracts were centrifuged at 15,000 *g*, at +4 °C for 20 min. The supernatants were diluted 10 times in 0.1 M potassium phosphate buffer; pH 8.0. To assess the glutathione amount, 100 µL of the extract and orthophthalic aldehyde (Fluka, Neu-Ulm, Germany) were introduced together into a quartz cuvette of 3 mL to a final concentration of 0.3 µm. The mixture was incubated in the dark for 15 min. Glutathione was assayed by recording the emission spectrum in the wavelength range of 350–500 nm upon irradiation at a wavelength of 290 nm using a Shimadzu RF 5301 PC spectrophotometer (Shimadzu, Tokyo, Japan). The reduced glutathione amount was calculated using the area of the emission spectrum curve with the rectangle method. To assess the oxidized glutathione, the initial extracts were diluted 10 times in 0.1 M NaOH with the addition of 1.6 mM N-ethylmalemide (Sigma-Aldrich, USA, St. Louis, MO, USA) before the measurement. The mixture was incubated for 30 min to stabilize the oxidized form of glutathione. To measure the level of the reduced and oxidized glutathione, the calibration curves within concentrations of 1–10 micrograms/mL were designed in each series of experiments. The reduced glutathione (Serva, Heidelberg, Germany) and the oxidized glutathione (Reagent, Budapest, Hungary) were used as the standards. The obtained amounts were calculated per dry biomass [39].

### 2.12. Potential-Dependent Staining

Potential-dependent staining of mitochondria in the *Y. lipolytica* cells raised at different pHs was performed using MitoTracker™ Red (Thermo Fisher Scientific, Waltham, MA, USA) as described before [40]. The red-stained preparations, filters 02, 15 (Zeiss, Oberkochen, Germany) were used (magnification ×100). Photos were captured using an AxioCam MRC camera (Zeiss, Oberkochen, Germany).

### 2.13. Transmission Electron Microscopy (TEM)

The TEM microscopy of *Y. lipolytica* yeast cells was performed as described before [39]. The sections were stained with uranyl acetate for 60 min and post-stained as described previously and examined with a Jeol (JEM-100B, Tokyo, Japan) and Hitachi U-12 electron microscopes (Hitachi, Tokyo, Japan).

### 2.14. Assay of the Protein Amount 

Total protein was assayed by the biuret method [41] and Bradford one with BSA as a standard [42]. The optical density of solutions was assayed on a spectrophotometer at 595 nm. 

### 2.15. Statistical Analysis

The experiments were performed in three independent biological triplicates with a standard error of less than 5%. The impact of pH was estimated using one-way ANOVA (n = 3) with R (R Core Team 2016). The Student’s *t*-test was used to analyze the significance of differences for independent samples. The means were considered significant at *p* < 0.

## 3. Results and Discussion 

### 3.1. Dynamics of Y. lipolytica Growth at Different pH 

**Figure 1:**
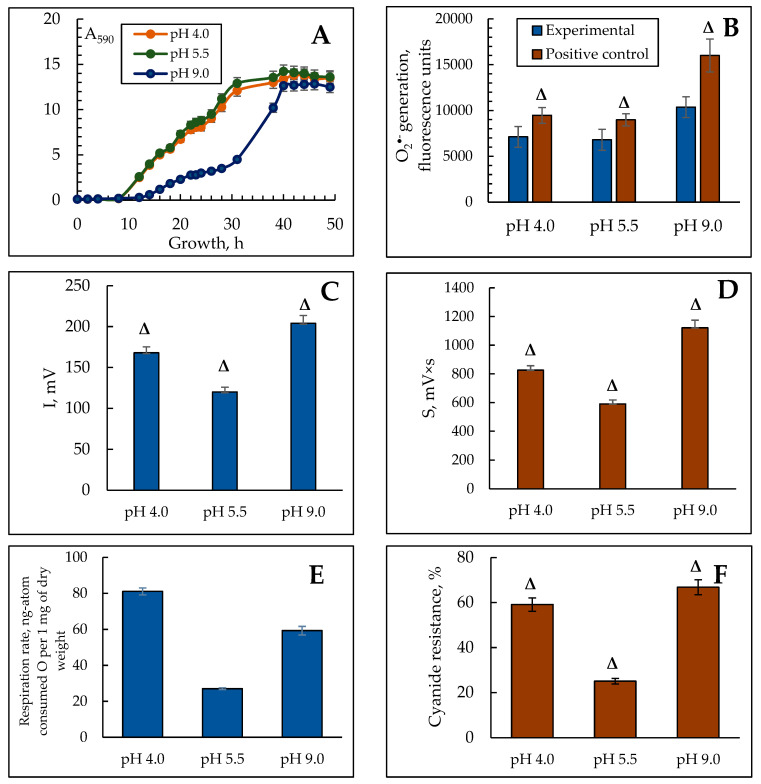
The effect of ambient pH on the growth (**A**), total ROS generation (**B**), BCL intensity (**C**,**D**) respiratory activity (**E**), and alternative oxidase induction (**F**) in *Y. lipolytica W29* yeast grown at various pHs. (**A**)—absorbance was assessed in cell suspension at the wavelength of 590 nm (A_590_); (**B**)—fluorescence intensity (units), reflecting total ROS generation in 60 min after the cell staining with H_2_DCF-DA at various pHs (blue columns). Cells exposed to pro-oxidant 600 μM AAPH were used as the positive control (brown columns). The incubation medium for the experiments contained 50 mM KPi, and 1% glucose, pH 5.5. Δ—statistically significant difference compared to the corresponding control, *p* < 0.005. Values are mean ± S.E.M from 5–6 independent experiments.

We have previously shown that the *Y. lipolytica* yeast optimum pH is within the typical range for the yeast range, namely of 4.5–6.0 [43]. The growth curve at different pHs from 3.5 to 4.5 showed a gradual rise, with a slight increase at a pH of 5.0 and a slight decrease in cell accumulation at a pH of 10.5–11.0. A change in growth conditions to the alkaline pH (pH ≥ 7.5) decreased a specific growth rate (µ) by four times; however, the longer logarithmic growth stage permitted to raise the cell mass comparable to that under the optimal conditions. Thus, we can attribute the *Y. lipolytica* W29 strain to moderate alkalotolerant yeast [44]. But the optimum pH of 5.5–6.0 typical for most yeast can be considered optimal for *Y. lipolytica*, too. The biomass yield decreased by 20% and 60% while cultivating at pH 4.0 and 9.0, respectively. The graph of the ambient pH on the biomass yield is shown in Figure 1A and reflects a complete image of the yeast accumulation up to the stationary phase. It is seen from the figure that the changes in pH affect the duration of the lag period and the exponential growth phase of the population. The lag period at pH 4.0 and 5.5 lasted less than 8 h, while at pH 9.0 it elongated up to 13 h. Moreover, at pH 9.0, we observed some slowdown in the culture growth rate that stretched the logarithmic phase (Figure 1A). The transition to the stationary growth phase at an alkaline pH happened 10 h later than that at pH 4.0 and 5.5. Evaluation of growth parameters indicated the pronounced stress in the culture at extreme ambient pH, especially at an alkaline pH. 

### 3.2. The ROS Generation in the Y. lipolytica Population Under pH Stress 

Any stress on the cells leads to, finally, oxidative stress, which is a critical imbalance between the production and detoxification of the ROS. Superoxide anion radicals are the most dangerous ROS in the *Y. lipolytica* cells, which we assayed at different pHs. The cells exposed to 600 µM 2,2′-azobis(2-methylpropionamidine) dihydrochloride (AAPH) were used as the positive control. Figure 1B shows the results. The level of the cell ROS generation in the population grown at alkaline pH was 1.5 times higher than that in the control. In the population grown at acidic pH, it changed slightly. The positive control containing 600 µM AAPH showed a similar level of the ROS production (Figure 1B). 

Maintaining the native conformation of the membrane structures based on lipid homeostasis is known to be the main adaptive feature of yeast under various kinds of stress [45]. The oxidation of unsaturated lipids of cell membranes induced by ROS (lipid peroxidation) causes, in turn, some changes in membranes, distorting cell physiology, up to its death [46]. Moreover, the stresses of various nature (heat shock, substrate depletion, salt, osmotic, and oxidative stresses) inhibit protein synthesis, in particular, at the stage of elongation and termination, which also affects the cell membrane functions [47]. It has also been shown in *Y. lipolytica* in response to osmotic stress [48]. It is noteworthy to say that the main active ROS, including some intermediates, are short-lived compounds, and difficult to detect. Consequently, most biochemical approaches to assessing the intensity of free radical oxidation processes are indirect. A more accurate methodological approach is the assay of biochemiluminescence, which permits real-time assessment of the lipid radicals level upon detecting their glow in the visible spectrum in spontaneous and iron-induced modes. The method is based on the Fenton reaction. Additionally, the biophysical technology helps clarify the current total antioxidant activity of the biosubstrate.

Using the method of iron-induced biochemiluminescence, we also assayed the intensity of free radical processes in the *Y. lipolytica* yeast upon adapting to various pH conditions. We found that in the stationary growth phase of the *Y lipolytica* yeast BCL, Imax, and S, reflecting the intensity of free radical processes increased significantly at extreme pH (Figure 1C,D). Thus, the intensity of the maximum flash 1.4- and 1.7-fold increased, respectively, at a pH of 4.0 and 9.0 compared to that at pH 5.5 (Figure 1C). However, the flashlight sum, significantly increased by 1.4 and 1.9 times under extremely acidic and alkaline conditions, respectively (Figure 1D). Along with this, the high adaptive ability of yeast and fungi is reported to be associated with a full complex of antioxidant protection, which prevents oxidative stress in the cells [49,50]. Upon studying the tangent of the incidence angle of the kinetic curve determining the total antioxidant activity, it was found that it was 1.2 times higher at a pH of 4.0 than at both pHs of 5.5 and 9.0, which practically did not differ (Table 1). 

The results on the induction of free radical oxidation in the *Y. lipolytica* cells under extreme conditions agree well with the reference data, namely, the assay of the ROS generation rate in the *Y. lipolytica* homogenates revealed a significant increase in the ROS production at both acidic and alkaline pH, whereas, at an optimal pH, the ROS generated a 1.5-fold decreased [51]. The highest antioxidative potential is awakened under acidic pH, which complies with the previous notions on the acid stress mechanism. 

### 3.3. Respiratory Activity of the Y. lipolytica Population at pH Stress 

The free radical production is tightly coupled with the cell aerobic metabolic activity, namely, cellular respiration. Two main enzyme systems transferring the electron through the inner membrane of the mitochondria, namely the cytochrome and cyanide-resistant pathways, contribute to the total oxygen consumption in some eukaryotes (yeast, fungi, and plants) [52]. The share of the alternative oxidase to the total rate of cell respiration can be considered as the most convincing marker for oxidative stress, since the ROS lead to peroxidation of cardiolipin, obligatory for the functioning of the cytochrome oxidase. Exposing the population with KCN establishes a similar situation. Consequently, the cell has to switch to an alternative oxidation pathway [53]. The results of the cell respiration rate and the share of the alternative oxidase to it are shown in Figure 1E,F. The most active respiration in the culture grown at pH 4.0 and 9.0 is exceeded by three- and two-fold, respectively, compared to that at normal pH (Figure 1E). However, the activity of the alternative oxidase nearly doubled at any stress reaching almost 70% of the total cellular respiration under the alkaline conditions (Figure 1F). It and the reduced specific growth rate could indicate severe oxidative stress and decreased metabolic activity. It is noteworthy that an increase in the respiration rate accompanied by an increased proportion of cyanide-resistant pathways was reported in 1999 for *Y. lipolytica* [54]. The switch of the respiration to an alternative pathway is less effective in ATP synthesis, but not in oxygen consumption. Considering that there are at least nine alternative oxidase genes for *Y. lipolytica* (*AOX*, *CJU89_1496*, *CKK34_6440*, *CKK34_4427*, *CJU89_4770*, *YALI1_D12567g*, *B0I71DRAFT_134507*, *YALI1_E01213g*, *YALI0_D09933g*) [55], the induction of an alternative pathway either at stress or upon inhibiting the cytochrome pathway undergoes fast enough to decrease the oxygen consumption rate and sometimes to increase it [56]. Thus, an assay of the redox and energy status of the *Y. lipolytica* population at acidic and alkaline pHs demonstrated the evident adaptive reply of the culture to stress.

### 3.4. Ultrastructural Features of the Y. lipolytica Yeast Cells at Different pH 

**Figure 2:**
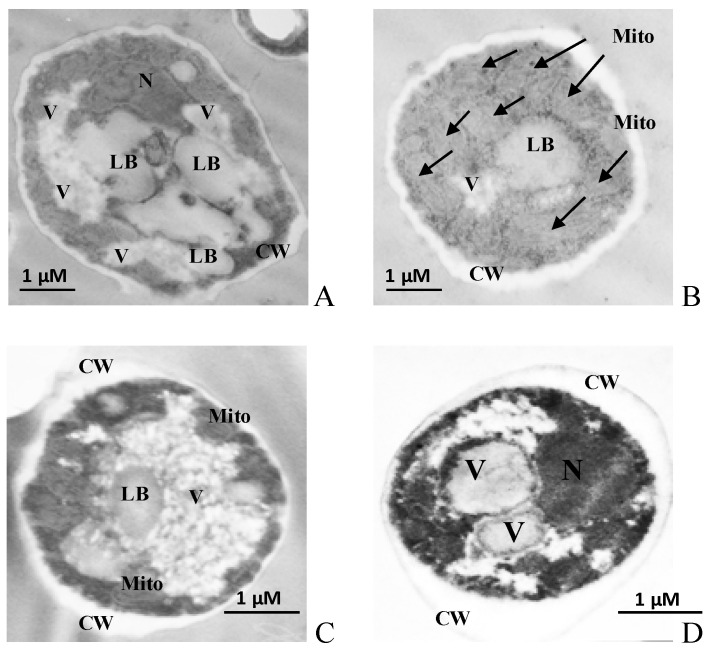
Ultrastructure (**A**–**D**) of the *Y. lipolytica W29* cells. (**A**,**B**)—pH 5.5; (**C**)—pH 4.0; (**D**)—pH 9.0. N—nucleus, Mito—mitochondria, V—vacuole, LB—lipid bodies, CW—cell wall. Mitochondria are also indicated by arrows.

Stress on the cell leads not only to changes in the growth and energy features of the cell but also to various reversible and irreversible changes in the cellular ultrastructure. It happens due to the alterations of the cell membrane structures, which can either serve as reliable markers of the cell physiological state or indicate the transition of the cell to apoptosis. Based on the data, we studied the ultrastructure of *Y. lipolytica* cells grown under various conditions using TEM. The micrographs of cells are shown in Figure 2. The figure with the ultrastructure of *Y. lipolytica* cells shows that all the microimages had a clealy visualized nucleus, mitochondria, and lipid inclusions. However, the cells grown under optimal conditions (Figure 2A,B) were large (7–10 µm) compared to the cells grown at extreme pHs (Figure 2C,D). The cells grown under optimal conditions had a lot of lipid droplets with low electron density, thin cell walls, and minimal vacuolation (Figure 2A,B).

The lipid bodies are known to play a key role in lipid metabolism and protein circulation, maintaining cell energy homeostasis. In [57], a stress-induced metabolic transition from fermentation to respiration with the promotion of peroxisomal β-oxidation of fatty acids was described. It is assumed that switching to alternative energy sources (in this case, storage lipids) promotes some signaling events demonstrating damage to the mitochondria at stress. Probably, in the case of pH stress in our yeast model, the mechanism also acts. 

**Figure 3:**
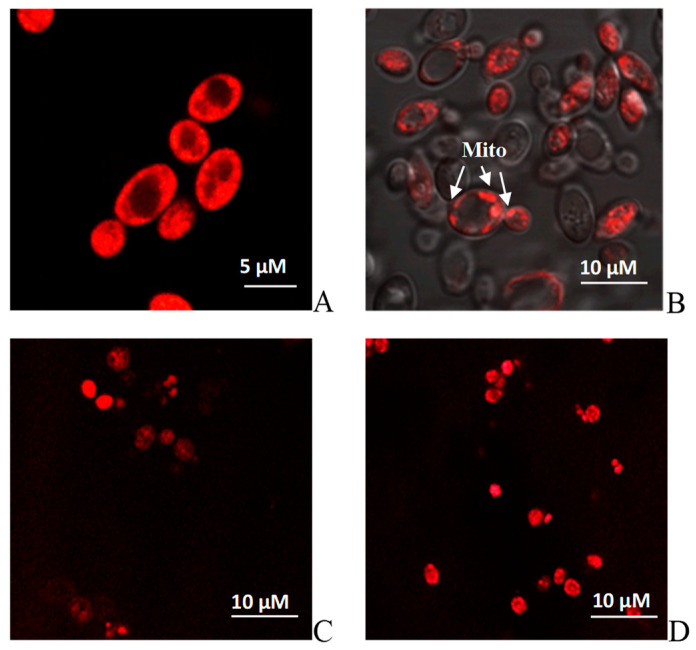
Potential-dependent staining of the mitochondria in *Y. lipolytica W29*. (**A**,**B**)—pH 5.5; (**C**)—pH 4.0; (**D**)—pH 9.0. Cells were incubated with 0.5 µM MitoTracker Red for 20 min. The incubation medium contained 0.01 M PBS, 1% glycerol, pH 7.4. The areas of high mitochondrial polarization are indicated by bright-red fluorescence due to the concentrated dye. To examine the MitoTracker Red stained preparations, filters 02 and 15 (Zeiss) were used (magnification 100×). Photos were captured using an AxioCam MRc camera.

In addition, the ultrastructure analysis showed that the cells grown under all the tested conditions had a dense cell wall with noticeable seals in the scar areas, especially pronounced at pH 4.0 and 9.0 (Figure 2C,D), which is, apparently, a protective reaction to stress. In all the cases, the cytoplasmic membrane formed deep invaginations. However, the cells grown at extreme pH showed noticeable vacuolization in the cytosol. Yeast vacuoles are complicated organelles involved both in the enzymatic protein degradation and the homeostasis of soluble compounds, namely, ions, sugar alcohols, and amino acids [58]. Of particular interest is the capability of yeast cells to accumulate some proline, an amino acid protecting cells against various stress, namely, heat shock, dehydration, and oxidative stress [58]. Proline can not only stabilize membrane structures but also intercept the ROS. So, in [59], the protective function of vacuoles as proline cellular depot was shown at various stresses using the *S. cerevisiae* cells. It is notable that the cytoplasmic space of the *Y. lipolytica* cells was rich in well-developed mitochondria of the near-wall location, with a pronounced cristae structure (Figure 2B). Potentiometric staining of cells with MitoTracker™ Red dye demonstrated a high induction of yeast mitochondria under both optimal (Figure 3A,B) and stress conditions (Figure 3C,D), which agreed well with the high respiration rate of the *Y. lipolytica* cells reflecting high metabolic activity at extreme pH.

### 3.5. The Assessment of the Activity of the Tricarboxylic Acid Cycle Enzymes at Extreme pH

Using the proteomic methods for the *Y. lipolytica* population, it was shown that all the proteins with the increased amount at extreme pH compared to those at a normal one, were the proteins of mitochondrial location and directly involved in the energy metabolism, namely, Krebs cycle enzymes, a-ketoglutarate dehydrogenase [29,60], and malate dehydrogenase [29,30], outer membrane protein (porin VDAC, non-selective voltage-gated ion channel), and the respiratory chain components [29,30,31]. Based on the data on the high energy status of the *Y. lipolytica* cells confirmed by their ultrastructure, we assessed the activity of some enzymes of the tricarboxylic acid cycle, namely NAD-IDH, AH, NAD-MDH, and FH.

#### 3.5.1. NAD-IDH

**Figure 4:**
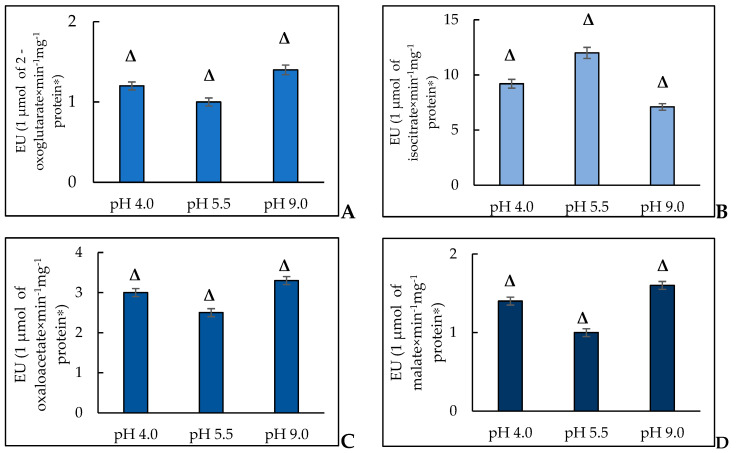
The activity of NAD-IDH (**A**), AH (**B**), NAD-MDH (**C**), and FH (**D**) in the *Y. lipolytica* cells grown at pH of 4.0, 5.5, and 9.0. * A unit of enzymatic activity (EU) is calculated as the amount of enzyme needed for 1 μmol of the final product for 1 min at a temperature of 25 °C. Δ—statistically significant difference compared to the corresponding control, *p* < 0.005. Values are mean ± S.E.M from 5–6 independent experiments.

In *S. cerevisiae*, there are two isoforms of NAD-IDH, namely, mitochondrial IDH1 (isocitrate dehydrogenase [NAD] subunit 1, mitochondrial) and IDH2 (isocitrate dehydrogenase [NAD] subunit 2, mitochondrial). NAD-IDH is an allosteric enzyme, which consists of two subunits localized in the mitochondria. For the *Y. lipolytica* strain Y-2373, NAD-IDH was described with a molecular weight of the native enzyme and subunits of 412 and 52 kDa, respectively [61]. The authors concluded that the enzyme is a homopolymer consisting of eight subunits. NAD-IDH is known to be active at high AMP levels and low NADH/NAD^+^ ratios. It has also been proven that high ATP and NADH levels inhibit enzyme activity [62,63,64]. The enzyme activity is provided by the sulfhydryl groups, namely, one SH group is located in the active center, and the other one maintains the active enzyme conformation. According to the obtained data, the specific activity of NAD-IDH while cultivated at a pH of 4.0 increased by 1.1 times compared to that at a pH of 5.5 (Figure 4A). Nevertheless, the growth of *Y. lipolytica* in extremely alkaline conditions was accompanied by a more significant increase in the specific enzyme activity (by 1.4 times) compared to that at a normal pH (Figure 4A). 

In paper [61], the authors reported that the citric acid overproduction by the *Y. lipolytica* cells is caused by the inhibition of NAD-dependent IDH as a fine regulator of citrate production by citrate synthase. Using *Debaryomyces hansenii*, a halotolerant/halophilic yeast, the effect of osmotic stress with NaCl (0.5 or 1.5 M) and LiCl (0.1 or 0.3 M) on the central carbon metabolism was assayed. The authors showed that the cells incubated with lithium similar to that for *Y. lipolytica* displayed higher isocitrate dehydrogenase activity and increased neither the activity of isocitrate lyase and malate synthase nor the transcription of the corresponding genes [65]. It was also shown that the salt increased the respiratory metabolism of *D. hansenii* [66]. Using the same strain, microarray-based gene expression assay in the cells incubated under the saline conditions and at high pH revealed the induction of some catabolic pathways, namely, the pathway of galactose metabolism, degradation pyruvate, and fatty acids. Moreover, the authors manifested a decrease in the expression of the genes associated with the energy production pathways, in particular, *DEHA2D15312G* (NADH-ubiquinone oxidoreductase) and *DEHA2C08448G* (ubiquinol-cytochrome-c reductase) [67].

The activation of NAD-IDH at extreme pH can be a universal response of the yeast cell to stress. Thus, in ref. [68], isocitrate dehydrogenase 1 (IDH1) could participate in retrograde signaling in osmo-stress as it is the key player in a rapid adaptive response to stress, which is in good agreement with our assumption. 

#### 3.5.2. AH

In higher eukaryotes, there are cytosolic (ACO1, Cytoplasmic aconitate hydratase) and mitochondrial (ACO2, Aconitate hydratase) forms of AH differing in physical-chemical and structural features. The iron–sulfur clusters (4Fe-4S) of the two AH isoenzymes are bound with cysteine residues of Cys437, Cys503, and Cys506 [69]. AH, with a cluster of 4Fe-4S in the structure, is subjected to cellular radicals. Currently, AHs are reported to be the main targets for ROS and nitrogen (RNS) species, namely, superoxide radical (O_2_^•–^), hydrogen peroxide (H_2_O_2_), nitric oxide (•NO), and peroxynitrite (ONOO^−^). In the case of ACO1, it was noted that H_2_O_2_ and •NO facilitate the activation of iron-sensitive proteins in the cells in vitro. AHs are also the main targets for cellular radicals both in the models and in vivo demonstrating such posttranslational oxidative modifications as S-nitrosylation and carbonylation of proteins [70]. 

The study of the AH activity, a marker of oxidative stress, revealed the induction of free radical processes in the *Y. lipolytica* yeast growing at extreme pH. Thus, in the *Y. lipolytica* population at optimal pH, the enzyme activity, expressed as a specific activity, was 1.7 times and 1.3 times higher than that at pH of 9.0 and 4.0, respectively (Figure 4B). The data have been confirmed by some other studies. So, in the *S. cerevisiae* yeast, the knockout in the *ACO1* (Δaco1) gene encoding the cytosolic enzyme isoform led to the damage of mitochondrial DNA [71] that permitted to assume that AH can directly interact with DNA, maintaining its stability [72]. In the study, it was shown for *Schizosaccharomyces pombe* that ACO2 is bound to the mRNA of iron uptake carriers controlling the homeostasis at the genetic level [73].

Recently, using confocal microscopy and cell sorting with fluorescence induction it was shown that at nitrosative stress the *ACO2* gene expression decreased. It was also noted that some of the enzymes of the tricarboxylic acid cycle were partially inhibited, whereas malate metabolism and alcoholic fermentation increased [74]. Some recent studies have confirmed the sensory role of AH upon the prooxidant action [75,76,77,78]. The results of some studies showed that the acidic pH decreases the level of the proteins containing iron–sulfur clusters, which are encoded by the *LEU1* and *ACO1* genes. Consequently, probably, we could suppose the AH sensory role under the oxidative stress conditions due to extreme pH [79].

Suppression of AH activity in response to pH stress deserves a separate comment. According to the reference, there are some ways of the AH regulating the activity, i.e., induction/inhibition resulting from assembly or disassembly of an iron–sulfur cluster or substitution of iron with any other metals; blocking due to the transformation of cysteine and tyrosine residues; competitive inhibition by di- and tri-carboxylic acids. The control of the enzyme expression can also occur at the post-transcriptional level [80]. Previously, we obtained data indicating some changes in the metal metabolism at pH stress, which could negatively affect the AH activity [35]. A similar phenomenon has also been described for the yeast cells at acidic stress conditions. In the studies using the AH purified preparation we showed that H_2_O_2_ decreased its activity [35]. Since the increased ROS production at alkaline pH (Figure 1B–D) for the *Y. lipolytica* yeast, an increased free radical level at pH stress is supposed to inhibit the AH activity. Moreover, the tight interaction of AH with NADP^+^-dependent IDH closely related to the enzymatic transformations of GSSG and GSH drags the enzyme into the interaction with the glutathione system and NADPH-supplying enzymes which are obligatory to function actively [81,82]. 

#### 3.5.3. MDH

**Figure 5:**
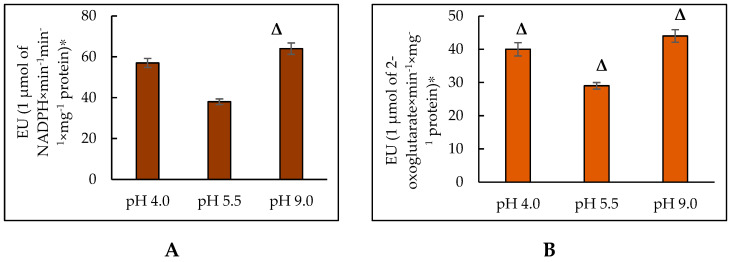
The activity of NADPH-producing enzymes in the *Y. lipolytica* yeast at extreme pHs. (**A**)—G6PDH; (**B**)—NADP-IDH. * The amount of enzyme catalyzing the formation of 1 μmol of the reaction product in 1 min at a temperature of 25 °C was taken as a unit of enzymatic activity (EU). Δ—statistically significant difference compared to the corresponding control, *p* < 0.005. Values are mean ± S.E.M. from 5–6 independent experiments.

MDH isoenzymes catalyze the conversion of malate to oxaloacetate with the NAD^+^ reduction [83]. The reversible reaction is an important step in the tricarboxylic acid cycle, a central metabolic pathway crucial for cellular respiration and ATP production undergoes in the mitochondria. MDH is also involved in gluconeogenesis in the cytosol and plays an essential role in the shuttle of malate/aspartate through the mitochondrial membrane [84]. Moreover, the enzyme is one of the most common population-genetic markers [85]. *S. cerevisiae* is known to possess three forms of MDH, namely, MDH2 (Malate dehydrogenase, cytoplasmic), MDH1 (Malate dehydrogenase, mitochondrial), and MDH3 (Malate dehydrogenase, peroxisomal). By contrast, the oleaginous yeast of *Y. lipolytica* contains only two MDH genes, namely, *YALI0D16753g* (*YlMDH1*) and *YALI0E14190g* (*YlMDH2*). Whereas the first gene encodes a protein of mitochondrial location, the second one does a putatively cytosolic form [80]. In our study, we found that the yeast growth at pH 4.0 and 9.0 was accompanied by an increase in the NAD-MDH activity by 1.2 and 1.3 times, respectively (Figure 5A). In S. *cerevisiae* yeast, the level of NAD(H)-specific IDH activity and immunoreactivity are reported to closely correlate with the level of MDH, the second enzyme of the tricarboxylic acid cycle [86]. In *S. cerevisiae* under all metabolic conditions, which need the the tricarboxylic acid cycle, namely, growing on “oxidative” substrates of glycerol and lactate, IDH and MDH were assayed at a constant level. We can suppose that couple induction of both enzymes also undergoes in the *Y. lipolytica* yeast (Figure 4A,C). In addition, it has also been previously shown that the altered features of the MDH oxidized forms promote the additional functions linking the energy metabolism with adaptive reactions needed to maintain redox homeostasis [87,88]. However, it is known that in plant cells, to resist oxidative damage, the flows of reducing equivalents are regulated by MDH isoforms in the corresponding cell compartment to maintain redox homeostasis. Thus, redox-dependent reversible inactivation and covalent dimerization appear in the *Arabidopsis* cells when the cytoplasmic isoform of MDH1 is oxidized [89]. It has also been shown that nuclear translocation of MDH causes transcription of the enzymes obligatory to overcome cadmium stress in the *Candida tropicalis* yeast [90]. Based on the facts, MDH in *Y. lipolytica* is supposed to play the role of some sensor maintaining redox homeostasis in the yeast cell upon adapting to extreme pH. 

#### 3.5.4. FH

FH belongs to the class of lyases and performs a reversible conversion of fumarate to L-malate [91]. FH of the yeast possesses two forms, namely, mitochondrial (FUM1, Fumarate hydratase, mitochondrial) and cytosolic (FUM2, Fumarate hydratase, cytosolic) ones, being very close in amino acid sequence, which is encoded by the nuclear genome [92]. In our study, we revealed that cell growth at both acidic and alkaline pH was accompanied by an increase in the fumarase-specific activity (by 1.4 times and 1.6 times, respectively) (Figure 4D). More and more papers suggest the possibility of the additional nuclear function of cytosolic fumarase. Nuclear recruitment of fumarase has been found to occur upon damage to double-stranded DNA in both yeast and human cells. Inside the nucleus, fumarase converts malate to fumarate, and its enzymatic activity plays a crucial role in the response to DNA damage [93,94]. Fumarase provides the protection function by directly binding to Nfs 1 in the mitochondria [95]. The yeast FH is also capable of modifying its functions upon posttranslational changes [96]. In our conditions of stress-induced hyperoxidation in the *Y. lipolytica* population, it is most likely that FH can act as a modulator of oxidative metabolism. Thus, based on the activities of the key enzymes of the Krebs cycle, a change in their activities in the mitochondrial matrix is supposed to be involved in the regulation of the cell metabolism of *Y. lipolytica*, which lets this species grow well at extreme pHs. It distinguishes it from any other type of ascomycete. The activity of enzymes can alter both due to regulatory mechanisms and the changes in the enzyme amount. 

### 3.6. Assay of the Activity of NADPH-Producing Enzymes in the Y. lipolytica Cells at Extreme pH

The expeditious functioning of the antioxidant system is of great importance in adapting to stress depends on the production of NADPH in the cell. The glutathione system, catalase, and superoxide dismutase are the three main components of the enzyme system in the cells. The function of the glutathione system is determined mainly by the production of reduced glutathione by glutathione reductase, depending on the NADPH level. Catalase does not need NADPH to convert hydrogen peroxide into water, but has an allosteric binding site for NADPH supporting catalase in its active conformation. Superoxide dismutase uses no NADPH to convert superoxide to hydrogen peroxide, but if neither catalase nor glutathione adequately restores it chemically, the elevated levels of hydrogen peroxide increase even more and inhibit superoxide dismutase. Consequently, the whole antioxidant system depends on NADPH [97]. The NADPH amount in the cytoplasm depends on the activity of the pentose phosphate pathway, namely, its first enzyme of NADP-dependent G6PDH [98,99], and also the activity of some enzymes providing the alternative production of NADPH, in particular, NADP-dependent IDH [100].

G6PDH catalyzes the conversion of glucose-6-phosphate to 6-phosphoglucono-σ-lactone with NADP^+^ as an electron acceptor. The equilibrium of the reaction is strongly accented towards the NADPH formation. In animal cells, G6PDH is reported to play an essential role in cellular survival and the prevention of ROS-induced cell death [94,98]. However, the data on the G6PDH activity dynamics in the yeast cells are limited. NADP+-dependent IDH belongs to the class of IDHs, which catalyze the oxidative decarboxylation of *D*,*L*-threo-Ds-isocitrate into 2-oxoglutarate. Participation in the cell biosynthetic processes is the main NADP+-dependent IDH function [81]. The oxidation of isocitrate to 2-oxoglutarate, catalyzed by NADP-IDH, is closely coupled to the enzymatic transformations of GSSG and GSH (2GSH = GSSG + 2H^+^), induced by GLR. NADP^+^-dependent IDH isolated from *Y. lipolytica* CLIB122 (YlIDP) is an enzyme with absolute specificity to NADP^+^, with K_m_ for isocitrate of about 60 µM [99]. The assay of the activity of G6PDH in the *Y. lipolytica* cells revealed that in the early stationary growth phase, upon being cultured at a pH of 4.0, the specific activity of the enzyme was 1.5 times higher compared to that at a pH of 5.5. Upon cultivation at extremely alkaline pH, the enzyme activity significantly (1.7 times) increased compared to that under the normal conditions (Figure 5A). However, the specific activity of NADP-IDH increased much less, namely by 1.4 times at pH 4.0 and 1.5 times at pH 9.0 compared to that at pH 5.5 (Figure 5B).

Damage of the *ZWF1* gene encoding G6PDH in the yeast creates phenotypes with relatively weak growth and increases the sensitivity to external oxidants, for example, hydrogen peroxide. The studies aimed at identifying other important sources of NADPH in the yeast showed a rapid loss of viability upon damaging the *ZWF1* gene and the *IDP2* gene encoding the cytosolic NADP-IDH. The results suggest that Zwf1p and Idp2p are obligatory sources of NADPH for thiol-dependent antioxidant reactions scavenging harmful byproducts of oxidative metabolism [97]. The NADPH depletion due to the *ZWF1* gene damage was reported to increase the tolerance of *S. cerevisiae* to both oxidative and nitrosative stress resulting from the conjugated activation of the cytosolic catalase of Ctt1. The authors showed that the NADPH-independent mechanism increases oxidative and nitrosative stress resistance in yeast cells without *ZWF1* [101]. 

Based on our results and reference data, we could conclude that the increased activity of NADPH-producing enzymes in the *Y. lipolytica* population at pH stress is an adaptive tool for overcoming oxidative stress. 

### 3.7. The Assay of the Enzymes of the Glutathione Antioxidant System in Y. lipolytica upon Cell Adaptation to Extreme pH

Most of the ROS are known to be neutralized before they destructively affect the cell. Thus, for one million superoxide radicals, there are only about four ions escaping the enzyme protection. Yeast cells possess an effective system of antioxidant enzymes and can scavenge the ROS maintaining the redox potential of cells [102]. Catalase, superoxide dismutase, thioredoxin (glutathione)–peroxidase, and peroxiredoxins are the key enzymes involved in ROS deactivation [103,104]. Yeast mitochondria contain Mn^2+^-dependent superoxide dismutases (SOD2), glutathione peroxidase, glutathione reductase, NAD(P)H-transhydrogenase [102]. The powerful ROS scavenging system in yeast cells protects the mitochondria from oxidative stress due to the damage and dysfunction in the cell. The first line of the antioxidant protection includes SOD and catalases [105,106,107,108]. According to the references, upon both adapting to extreme pH and being treated to high concentrations of some prooxidants, namely paraquat and hydrogen peroxide, at the increased oxygen, the *Y. lipolytica W29* cells showed the ROS-induced promotion of SOD, which is considered a universal mechanism for protecting the unique strain from unfavorable conditions [109,110]. Previously, we showed that the SOD activity during the cultivation of *Y. lipolytica* at pH 9.0 was four-fold higher than that at a pH of 5.5. Under the acidic conditions (pH 4.0), the SOD activity in the yeast cells was about 2.5 times that at a pH of 5.5. The activity of another antioxidant enzyme interacting with SOD, catalase, was maximal in the cells grown at an optimal pH, whereas at extreme pH, the enzyme displayed significantly lower activity [110]. We supposed that the obtained SOD activation is a universal adaptive response to stress in *Y. lipolytica* yeast. However, the reduced catalase activity may be due to the protein auto-inactivation at the increased ROS level as was shown before, both for the enzymes from animal tissues [111,112]. It was of particular interest to study the enzymes of the glutathione antioxidant system in the *Y. lipolytica W29* cells in their protection function from excessive hydrogen peroxide at the ROS-induced induction of the SOD upon adapting to extreme pH. 

**Figure 6:**
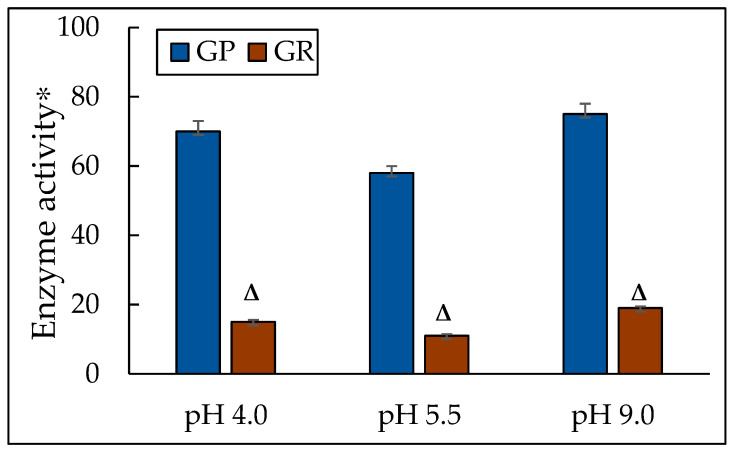
The activity of glutathione peroxidase and glutathione reductase in the *Y. lipolytica* culture grown at different pHs. * Unit of enzymatic activity per 1 mg of protein. Cellular GPxs were measured at 340 nm in 0.05 mM K-P_i_-buffer, pH 7.4; containing 1 mM EDTA, 0.12 mM NADP^+^, 0.85 mM GSH, 0.37 mM H_2_O_2_, and 1 unit of GR per mL. The same medium without glutathione served as the control. The reaction was initiated by the enzyme application. The rate of NADP^+^ reduction due to enzyme reactions such as oxidized glutathione formation by GPxs action followed by its reduction resulting from NADP^+^ oxidation by GR action was monitored by a decrease in A. One unit of enzyme (EU) activity was defined as the enzyme amount catalyzing 1 µmol final reaction product at +25 °C per min. The calculation is the same as that for IDH. Δ—statistically significant difference compared to the corresponding control, *p* < 0.005. Values are mean ± S.E.M. from 5–6 independent experiments.

The results of our studies showed that cell growth at both acidic and alkaline pH was accompanied by an increase in the glutathione peroxidase activity (by 1.2 times and 1.3 times, respectively) (Figure 6). Moreover, the glutathione reductase activity increased a bit more, namely, by 1.4 times at pH of 4.0 and by 1.7 times at a pH of 9.0 compared to those at pH of 5.5 (Figure 6). The activity of the GLR/GPx system is known to depend on GSH concentration. Therefore, we assayed the concentration of GSH in the cell homogenates (Table 2). The GSH level was 21-fold and 42-fold higher at pH 4.0 and 9.0, respectively than that at pH 5.5. We suppose that the constant GSH level in the yeast cells could be explained by the induction of not only GP but also regenerating GSH GLR. 

GPxs/GLP system being a universal and highly conservative disulfide reduction pathway includes a large family of GPxs detoxifying hydroperoxide-containing substrates via GSH, FAD-bound GLP, which reduces GSSG to GSH [113]. NADPH is the final donor for the systems. The GPs family containing eight selenium-dependent and “non-selenium” enzymes (GPx1–8), belonging to three large groups, catalyzes the reduction reaction of organic hydroperoxides and their alcohols via GSH [114]. GPs are of a wide substrate specificity, i.e., their substrates are organic hydroperoxides (cholesterol hydroperoxides, fatty acids) and thiol-containing compounds (dithiotreitol, glutaredoxin, thioredoxin, etc.) [114,115]. However, most GPxs are specific to GSH, which oxidizes during the reaction. The location, taxonomic affiliation, and functions of the enzymes vary widely [116]. Currently, GPxs are considered antioxidant agents of pivotal importance in protecting the cell from the generated hydroperoxyl radicals of various nature. Now, only one gene has been found in the genome of *Y. lipolytica* of high similarity to the GPx of *S. cerevisiae YIR037*. It is the YALI0E02310 protein [117]. Functioning GPx is tightly coupled with the GLR action, which regenerates GSH from GSSG due to NADPH-dependent reduction. GLR is an FAD-dependent homodimer with K_m_ for GSSG of 55 µm [118]. A gene encoding GLR—*GLR1*—has been identified in the *S. cerevisiae* yeast. The deletion of the gene decreased both GSSG and GSH levels, and the mutants were of a higher sensitivity to the oxidants [116]. In the genome of *Y. lipolytica*, one gene similar to *Glr1* from *S. cerevisiae*, namely the *YALI0E18029* gene, has been identified [119]. The induction of GLR is assumed to be one of the nonspecific cell responses to any stress. The enzyme could maintain the normal redox potential of glutathione in the cytosol [120] and mitochondria [121]. GLR deficiency leads to mortality of *Schizosaccharomyces pombe* and some congenital diseases in humans [122]. Nevertheless, the *S. cerevisiae* mutants in Δ*glr1* (devoid GLR) showed a high level of GSSG in the cytosol, but they did not differ in growth features from the wild type [120]. 

Our results agree well with the studies by Biryukova et al., where the authors showed the increased enzyme activity at various stresses using the *Y. lipolytica* cells. Thus, the authors manifested that the activity of enzymes against the oxidative stress, namely, catalase, superoxide dismutase, glucose-6-phosphate dehydrogenase, and glutathione reductase upon acting exogenous prooxidants H_2_O_2_ (0.3 mM), menadione (0.05 mM), and juglone (0.005 mM) in the yeast cells increased [123]. The treatment of the *Y. lipolytica* cells in the growth stationary stage with non-lethal doses of the stressors doubled the activity of GR [51].

Thus, the results confirm the leading role of the main NADPH-generating systems in the *Y. lipolytica* cells in responding to extreme pH-induced stress.

## 4. Conclusions 

**Figure 7:**
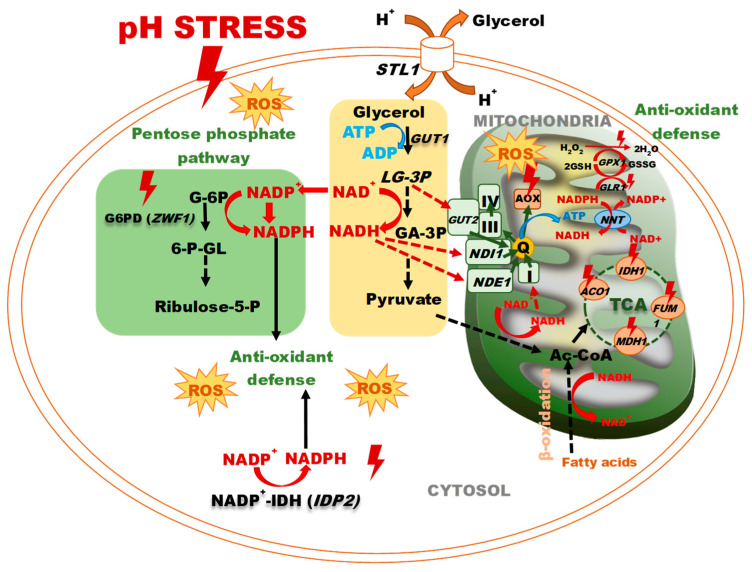
The response of a yeast cell to pH stress. Abbreviations: L-G3P: L-glycerol-3-phosphate, GA-3P: glyceraldehyde-3-phosphate, STL1: glycerol/H^+^ symporter, GUT1: glycerol kinase, G-6P: glucose 6-P, 6-P-GL: 6-P-gluconolactone, GUT2: FAD-dependent glycerol-3-phosphate dehydrogenase, MDH1: malate dehydrogenase, FUM1: fumarase, ACO1: aconitase, IDH1: isocitrate dehydrogenase, NADP^+^-IDH: NADP^+^- isocitrate dehydrogenase, G6PD: glucose-6-P dehydrogenase, Ac-CoA: acetyl-CoA, NDE1: mitochondrial external NADH dehydrogenase, NDI1: NADH: ubiquinone oxidoreductase, Q: ubiquinone, III: complex III, IV: complex IV, AOX—alternative oxidase, TCA: tricarboxylic acid cycle, GLR1: glutathione reductase, GPX1: glutathione peroxidase, NNT: NADP^+^-transhydrogenase.

Yeast is widely used as a model in the study of various physiological processes in eukaryotes at different stresses, from osmotic to thermal and oxidative stress. The most striking feature of a model of lower eukaryotes is the unique ability of *Y. lipolytica* not only to resist alkaline stress but also to grow well at a pH up to 10.5 [19,80]. In our study, we first thoroughly assayed the response of the key enzymes of the energy metabolism and the antioxidant system in *Y. lipolytica* yeast at extreme pH. We have demonstrated a close relationship between the induction of the Krebs cycle enzymes and the main enzymes of the glutathione system with the increased reduced glutathione. Maintaining the cellular redox balance between NAD^+^ and the reduced NADH may be crucial for many metabolic processes. It could be tested by triggering catabolic and anabolic reactions by NAD^+^ and NADH oxidation and reduction. The Krebs cycle of NAD-IDH and NAD-MDH mainly supplies NADH in the mitochondria. Then, NADH can be used either to produce ATP in the electron transfer chain or to be converted to NADPH upon the nicotinamide nucleotide transhydrogenase reaction. Moreover, NADPH is used to support the antioxidant systems of GP and/or peroxyredoxin (PRX) via the GR and/or thioredoxin reductase (TR) enzymes. Moreover, the promotion of NADPH-producing enzymes, namely, NADP-dependent G6PDH and NADP-IDH, contributes to the NADPH pool, which protects the yeast cells against oxidative stress at extremely acidic and extremely alkaline pHs. Thus, the increased activity of the Krebs cycle dehydrogenases and the activation of the pentose phosphate pathway at pH stress provokes some events determining the adaptive response of the *Y. lipolytica* yeast (Figure 7). The highest antioxidative potential is awakened under acidic pH, which complies with the previous notions on the acid stress mechanism.

Our results can be widely applied in the medical and biotechnological fields. Thus, the oncogenic mutations in the cytosolic IDH (IDH1) have been detected in some cancerous neoplasms, namely, acute myeloid leukemia and poorly differentiated and secondary glioblastoma [124,125], cholangiocarcinoma, chondrosarcoma, and glioma [126]. In all the cases, the neomorphic activity of the mutated enzyme leads to the formation of the oncometabolite of D-2-hydroxyglutarate with profound cell-autonomous and non-cell-autonomous effects. A rapidly developing therapeutic approach is aimed at designing small molecule inhibitors targeting the mutant IDH1 (mIDH1). It could be confirmed by the creation of the mutant AG-120, being a selective inhibitor of the IDH1 [127]. *Y. lipolytica* as a model provides a magnificent approach to solving the problem. In this aspect, the study of stress-induced activity of IDH can significantly contribute to the studies. It should also be noted that mitochondrial NADH is tightly related to the biosynthesis of lipids and citric acid in oily yeast [99]. The enzyme activity is finely regulated by the cultivation conditions. In particular, nitrogen starvation leads to the accumulation of excessive amounts of cytosolic citrate, which is used for the biosynthesis of fatty acids and extracellular secretion of citric acid. It could allow for the consideration of mitochondrial NAD-IDH as a target in the genetic engineering of oily yeast.

## Figures and Tables

**Table 1:** Total antioxidant activity expressed as the tangent of the inclination angle of the kinetic curve of biochemiluminescence in the *Y. lipolytica* homogenates at different pHs.

Conditions	tg α *
pH 4.0	79 ± 3.87
pH 5.5	65 ± 3.25
pH 9.0	67 ± 3.34

* Note: Table discusses statistically significant differences compared to the corresponding control, *p* < 0.005. Values are mean ± standard deviation from 3 independent experiments.

**Table 2:** The amount of the reduced glutathione in the *Y. lipolytica* cell homogenates at different pHs.

Conditions	[GSH], mmols/L *
pH 4.0	0.29 ± 0.003
pH 5.5	0.24 ± 0.003
pH 9.0	0.34 ± 0.003

* Note: Table discusses statistically significant differences compared to the corresponding control, *p* < 0.005. Values are mean ± standard deviation from 3 independent experiments.

## Data Availability

The original contributions presented in the study are included in the article, further inquiries can be directed to the corresponding author.
